# New Advances in the Determination of Free and Bound Phenolic Compounds of Banana Passion Fruit Pulp (*Passiflora tripartita*, var. Mollissima (Kunth) L.H. Bailey) and Their In Vitro Antioxidant and Hypoglycemic Capacities

**DOI:** 10.3390/antiox9070628

**Published:** 2020-07-17

**Authors:** Elisa Giambanelli, Ana Maria Gómez-Caravaca, Arancha Ruiz-Torralba, Eduardo Jesús Guerra-Hernández, Jorge Geovanny Figueroa-Hurtado, Belén García-Villanova, Vito Verardo

**Affiliations:** 1 Department of Analytical Chemistry, Faculty of Sciences, University of Granada, Avd. Fuentenueva s/n, 18071 Granada, Spain; elisagiambanelli@ugr.es; 2 Department of Nutrition and Food Science, University of Granada, Campus of Cartuja, 18071 Granada, Spain; ruizarancha@ugr.es (A.R.-T.); ejguerra@ugr.es (E.J.G.-H.); belenv@ugr.es (B.G.-V.);; 3 Departamento de Química y Ciencias Exactas, Universidad Técnica Particular de Loja, San Cayetano Alto s/n, 11-01-608 Loja, Ecuador; jgfigueroa@utpl.edu.ec; 4 Institute of Nutrition and Food Technology ‘José Mataix’, Biomedical Research Center, University of Granada, Avda del Conocimiento sn., 18100 Armilla, Granada, Spain

**Keywords:** fruit, *Passiflora*, bioactive compounds, HPLC-ESI-TOF-MS, flavan-3-ols, proanthocyanidins, DPPH, ABTS, FRAP, antihyperglycemic activity

## Abstract

Banana passion fruit (*Passiflora tripartite* L.H. Bailey) is a lesser known species of the genus *Passiflora*. This fruit typically grows in the Andean region of Ecuador and it is locally known as tumbo, taxo or curuba. The juice of this fruit is highly appreciated in South America. Extracts of banana passion fruit were characterized for their content levels of free and bound phenolic compounds by high performance liquid chromatography coupled to high resolution mass spectrometry detector (HPLC-ESI-TOF-MS). A total of 82 polar compounds classified as phenolic acid derivatives, organic acids, benzophenones, flavan-3-ols, flavonols and flavones were detected in the extracts. The total phenolic content was 2356 mg 100 g^−1^ dry matter, with the bound phenolic fraction representing 37.7% of total amounts. Flavan-3-ols, such as (epi)catechin, (epi)azfelechin and their derivatives, were the main phenolic compounds in the free phenolic fraction; however, phenolic acids represented the most abundant class of bound phenolic extracts. The antioxidant and hypoglycemic capacities reported for banana passion fruit were higher than for other fruits. To our knowledge, this is the first time that bound phenolic compounds have been described in banana passion fruit pulp.

## 1. Introduction

Tropical fruits, with their delicious taste, refreshing flavor and sweetness, are particularly appreciated by consumers. They are often processed into juices, puree, canned fruits, jam and dehydrated bars. In the last few decades, their popularity has increased around the world, especially in developed countries, because they are associated with health promoting compounds [1,2,3]. Several investigations carried out in the last few years have highlighted the fact that these fruits are important sources of antioxidants such as phenolic compounds, vitamins, carotenoids and minerals [1,2,3,4,5,6,7,8,9,10,11,12,13,14,15,16,17,18,19,20,21,22,23]. Their high phytochemical content contributes to their preventive effect against chronic degenerative diseases. 

Latin America hosts a high number of crops and substantial tropical fruit biodiversity; the genetic resources of this area, still poorly exploited, could be addressed in order to deal with challenges such as sustainable agriculture development and food security [4]. The Andean region of Ecuador has a wealth of underexploited native and exotic fruits, for which the local population still maintains traditional uses [5,6]. The genus *Passiflora*, comprising about 500 species, is the largest genus in the family of Passifloraceae [7]. The leaves of this genus are mainly used and exploited for their phytotherapeutic properties as anti-inflammatory, anxiolytic and sedative substances [8,9]. Within this genus, the passion fruit varieties of *Passiflora edulis* Sims F. *flavicarpa* Degener and *Passiflora edulis* Sims F. *edulis*, most commonly known as maracuyá and gulupa, are highly appreciated by consumers [10]. A lesser known species of the genus *Passiflora* is banana passion fruit (*Passiflora tripartita var. Mollissima* L.H. Bailey). This fruit typically grows in the Andean region, and it is locally known as tumbo, curuba or taxo. With the typical shape of a banana, the fruit can reach around 40–110 g in weight and 8–15 cm in length. The pulp is gelatinous, and it surrounds several small black seeds. Locally, it is mainly consumed as juice, and its unique taste could be of particular interest to be exploited in niche markets. 

Recently, leaves, pulp, seeds and edible parts of banana passion fruit have been investigated regarding their composition, especially their phytochemical content [11,12,13]. Previous studies demonstrated that banana passion fruit is particularly rich in phenolic antioxidants [14,15]. Contreras-Calderón et al. [16] found that banana passion fruits had the highest levels of antioxidant activity compared to other fruits, and this fact has been attributed to the presence of high amounts of phenolic compounds [12,13]. As a result, there are studies that have investigated the evolution of these compounds following the microencapsulation of the fruit’s pulp [15].

The presence of phenolic compounds in foods is commonly investigated to determine their putative protective effects against several chronic diseases, as also highlighted by several experimental studies [17,18,19]. Phenolics in fruits and vegetables occur as soluble conjugated and insoluble forms, covalently bound to sugar moieties or to cell wall structures. These forms have a different absorption pathway in the digestive tract, with bound phenolic compounds reaching the colon intact in order to fully release and exhibit their bioactivity [20]. Several studies have also demonstrated the significantly higher antioxidant capacity of the insoluble bound phenolics with respect to free and soluble conjugated phenolics [21]. Notwithstanding this, investigations carried out on the phenolic content of fruits are mainly addressed at characterizing free phenolic compounds, underestimating the extent to which the total phenolic content is the sum of free and bound phenolic compounds. 

To our knowledge, banana passion fruit has never been analyzed and characterized for its content of bound phenolic compounds before. Consequently, the main aim of this study was the identification and quantification of free and bound phenolic compounds in banana passion fruit pulp by high performance liquid chromatography coupled to high resolution mass spectrometry detector (HPLC-ESI-TOF-MS). Moreover, the fruit’s antioxidant and hypoglycemic capacities were also assayed.

## 2. Materials and Methods 

### 2.1. Chemicals

HPLC-grade acetonitrile, methanol, acetic acid, ethanol, hexane, ethyl acetate, diethyl ether and hydrochloric acid were purchased from Merck KGaA (Darmstadt, Germany). Hydroxide sodium was obtained from Fluka (Buchs, Switzerland). Double-deionized water with conductivity lower than 18.2 MΩ was obtained with a Milli-Q system from Millipore (Bedford, MA, USA). Ferulic acid, catechin, quercetin and rutin (Sigma-Aldrich, St. Louis, MO, USA) were used for the calibration curves.

### 2.2. Sample 

About 5 kg of *Passiflora tripartita var. Mollissima* fruits were purchased from a local market in Loja (Ecuador). Pulp was manually separated, immediately frozen in encoded plastic bags at −20 °C, and then freeze-dried (Thermo HETO, powerdry LYOLAB 3000; Waltham, MA, USA). Dried samples were ground to a fine powder in a blender mixer (Ika-Werke M20; Staufen, Germany) and stored at −18 °C until extraction.

Before the drying process, soluble solids (° Brix), pH and acidity were determined, and the results are shown in Table 1.

### 2.3. Free and Bound Phenolic Compound Extraction 

The free phenolic fraction of pulp was extracted with the protocol suggested by Gómez-Caravaca et al. [23]. Briefly, 0.5 g of the previously freeze-dried sample was extracted using 10 mL of ethanol/water (80:20, *v/v*), using an ultrasonic bath (15 min at room temperature). After this, the resulting sample was centrifuged (15 min at 1000 g). This extraction process was replicated twice more. The solvents of the three extractions were pooled, carried out to dryness in a rotavapor and re-dissolved in 3 mL of methanol/water (50:50, *v/v*). The bound polar fraction was obtained after alkaline hydrolysis of the remains from free phenolic extraction. Digestion of the samples was performed at room temperature with NaOH 2 M (100 mL) (20 h under nitrogen atmosphere). Then, HCl was used to reach pH 2–3 in a cooling ice bath, and 500 mL of hexane was added to eliminate the lipid fraction. Three extractions with 100 mL of diethyl ether/ethyl acetate (50/50 *v/v*) were conducted; the fractions were collected and evaporated to dryness. The obtained extract was re-dissolved in 2 mL of methanol/water (1:1 *v/v*). 

Finally, 0.22 μm PTFE syringe filters were used to filter the extracts, and they were kept at −18 °C. 

### 2.4. HPLC-ESI-TOF-MS Analyses

Free and bound phenolic compounds were determined using an ACQUITY UPLC M-Class liquid chromatograph from Waters (Waters Corp., Milford, MA, USA) that included a degasser, a binary pump, an oven and an automatic liquid sampler, and it was coupled with mass spectrometry. Phenolic compounds were separated on HPLC column Poroshell 120, SB-C18 (3.0 mm × 100 mm, 2.7 µm) from Agilent Technologies (Agilent Technologies, Palo Alto, CA, USA). Oven temperature was set at 25 °C and separation was conducted according to the conditions previously proposed by Gómez-Caravaca et al. [23]. 

### 2.5. Evaluation of Antioxidant Capacity

The antioxidant capacity of the extracts was evaluated using three assays, namely DPPH, ABTS and FRAP. The analyses were developed on Fluostart Omega microplate apparatus (BMG LabTech, GmbH, Ortenberg, Germany). The protocols used were the same adopted by Ruiz-Torralba et al. [24]. The results are the mean of three replications and are expressed as µmol Trolox equivalent (TE)/g of sample dry weight (d.w.).

### 2.6. In Vitro Hypolycemic Capacity via α-Amylase Inhibitory Assay

The α-amylase inhibitory test was carried out according to Leporini et al. [25], measuring the production of maltose after the incubation of starch with α-amylase (with and without *Passiflora* passion fruit extract). The extract was added to 3,5-dinitrosalicylic acid and the reaction mixture was measured at 540 nm.

### 2.7. Statistical Analysis

The results reported in this study are the averages of three repetitions (*n* = 3). Pearson’s linear correlations at the *p* < 0.05 level were evaluated using Statistica 6.0 software (2001, StatSoft, Tulsa, OK, USA).

## 3. Results 

### 3.1. Identification of Free and Bound Phenolic Compounds

Banana passion fruit is a popular fruit from Ecuador, especially appreciated for its juice. Free (FPC) and bound (BPC) phenolic compounds have been extracted from the pulp of this fruit and characterized by means of HPLC-ESI-TOF-MS (Figure S1). Extract ion chromatograms of the peaks, exact mass (*m/z*), molecular formula and fragment ions formed at ion source, together with retention time, Error, mSigma and literature, have been taken into account for identification purposes.

A total of 82 peaks have been detected and tentatively identified in banana passion fruit pulp extracts. Identified compounds belonged to organic acids, benzophenones and different classes of phenolic compounds; among these were flavan-3-ols, flavonols and flavones derivatives, phenolic acids and derivatives. All these compounds are reported in Table 2, together with the main information obtained by the HPLC-ESI-TOF-MS platform.

Peaks 1 and 2, with molecular formulas C_7_H_12_O_6_ and C_6_H_8_O_7_, were identified as organic acids, specifically quinic acid and citric acid, respectively. These compounds are commonly present in numerous fruits [23].

Several peaks have been identified as phenolic acids and derivatives, especially in the first part of the chromatogram. Peaks 3, 4, 6 and 7 showed the same fragment ion at *m/z* 191 that is due to the presence of quinic acid deprotonated moiety. In particular, peaks 3, 4 and 6 showed the same molecular formula, C_22_H_28_O_14_, and they were assigned to three derivatives of dicaffeoylquinic acid. The presence of dicaffeoylquinic acids and caffeoylglucaric acid derivatives (peak 79, with *m/z* 563.3261 and molecular formula C_24_H_52_O_14_) was already confirmed in passion fruit juice by Spínola et al. [26]. Peak 7, with *m/z* 527.1050 and molecular formula C_22_H_24_O_15_, was assigned to a caffeoyltartaric acid derivative, according to Spínola et al. [26], who isolated the same molecule in cherimoyas. Another hydroxycinnamic acid derivative was peak 63, which showed molecular formula C_18_H_16_O_8_ and *m/z* 359.0895 and which was tentatively identified as rosmarinic acid. This compound is a typical hydroxycinnamoyl derivative of Labiatae plants [27], but rosmarinic acid has also been isolated as a major compound, together with 3-*O*-caffeoylquinic acid, in *Solanum betaceum* Cav. cultivars from Ecuador [28]. Finally, peak 31 was identified as caffeic acid [11].

Among hydroxybenzoic acids, peaks 9 and 17 were respectively identified as 3,4-dihydroxybenzoic acid and 4-hydroxybenzoic acid. These phenolic acids have been recently found in the extracts of *Passiflora mollissima* seeds obtained by a pressurized liquid system and determined by UHPLC-Q-TOF-MS/MS analysis [11]. Peaks 10 and 23 were attributed to syringic acid, whereas peaks 69 and 81 were tentatively identified as syringic acid derivatives, specifically syringic acid hexoside and methylsyringin, respectively. Derivatives of syringic acid were previously detected in pineapple and mango by Septembre-Malaterre et al. [29]. Peaks 45 and 46, with molecular formula C_20_H_20_O_14_, *m/z* 483.1093 and a fragment ion at *m/z* 321.0748 due to the loss of a glucose moiety (M-162), were identified as two isomers of digalloylglucose; the same compounds were already identified by Gómez-Caravaca et al. [23] in mango fruits. 

Besides phenolic acids, a large number of flavonoids was also detected in banana passion fruit pulp extracts. Flavones and flavonols were mainly present as hexoside derivatives. Among these, peak 16, with molecular formula C_21_H_20_O_13_ and *m/z* 479.0881, was attributed to the flavonol myricetin hexoside, which has already been described in guava leaves [30]. Another myricetin derivative (peak 80) was tentatively identified, with molecular formula C_30_H_60_O_20_ and *m/z* 739.3630. This flavonol derivative was previously reported by Guimarães et al. [31] in wild fruits of *Prunus spinosa* from Northeastern Portugal. Peak 28, with molecular formula C_12_H_20_O_12_ and *m/z* 463.0841, was attributed to isoquercitrin, a glucoside of quercetin that has been isolated in other tropical fruits, such as mango and guava leaves [23,30]. Peak 62, with molecular formula C_28_H_32_O_17_ and *m/z* 639.1273, was assigned to isorhamnetin-*O*-dihexoside, in accordance with Spínola et al. [26], who determined for the first time the conjugate form of this flavonol in passion fruit and strawberry. 

Peak 50, with molecular formula C_15_H_12_O_6_ and *m/z* 287.0666, was identified as the flavanone eriodictyol, in agreement with previous HPLC–mass spectrometry screening of mango and its by-products [23]. Peaks 35 and 47, with molecular formula C_21_H_22_O_11_ and *m/z* 449.1234 and 449.1235, respectively, were tentatively assigned to isomeric forms of eriodictyol-*O*-hexoside, according to Pereira et al. [32]. Peak 70 was tentatively identified as phloretin dihexoside, showing a molecular formula of C_27_H_33_O_15_ and *m/z* 597.2018. These compounds have mainly been described in tomato samples [33]. Peak 74, with molecular formula C_30_H_26_O_10_ and *m/z* 545.1626, was also observed in extracts of *Passiflora mollissima* seeds by Ballesteros-Vivas et al. [11], and it was tentatively attributed to a trihydroxyflavanone dimer. 

Peak 36 showed a molecular formula C_21_H_20_O_11_ and *m/z* 447.0978, and it was identified as luteolin-6-C-glucoside (orientin). This flavone has already been reported in passion fruit of the genus *Passiflora edulis* by Spínola et al. [26].

Peaks 73 and 77, with molecular formula C_28_H_32_O_15_ and *m/z* 607.1625 and 607.1647, respectively, were identified as diosmetin-rutinoside. Peaks 43 and 54, with molecular formula C_28_H_32_O_16_ and *m/z* 623.1646 and 623.1641, respectively, were identified as diosmetin-dihexoside (lucenin-2,4-methyl ether). Diosmetin-hexoside derivatives have been reported in citrus fruits [26], and diosmetin-rutinoside has been detected in peel samples of *Passiflora mollissima* [34].

Peak 51, with molecular formula C_21_H_20_O_9_ and *m/z* 415.1068, was tentatively identified as daidzin. According to the literature, peak 76, with molecular formula C_22_H_20_O_9_ and *m/z* 427.1048, was assigned to durantin A; this compound was previously isolated and characterized by NMR in the whole plant of Duranta repens Linn, a subtropical species that is found in West Indies and South America [35]. Peak 56 showed a molecular formula of C_21_H_18_O_11_ and *m/z* 445.0916; a similar peak was identified as baicalin by Li et al. [36] in Scutellaria lateriflora, a plant that is popular for its mild relaxant properties and is used as a therapy for anxiety, nervous tension and convulsions. 

Peak 78, at *m/z* 557.1256 and molecular formula C_27_H_26_O_13_, was assigned to 2,6-dihydroxy-3-methyl-4-*O*-(6’’-*O*-galloyl-β-d-glucopyranosyl)-benzophenone. This compound has been previously isolated in Psidium guajava L. fruits and leaves [30,37].

Flavan-3-ols represented a numerous class of phenolics in banana passion fruit pulp extracts. Peak 27, with molecular formula C_15_H_14_O_6_ and *m/z* 289.0721, was assigned to catechin, and its identification was also confirmed by co-elution with a commercial standard. Several peaks of the chromatogram showed a fragment ion at *m/z* 289, meaning that they were (epi)-catechin derivatives. Peaks 37 and 38, at *m/z* 435.1230, showed a loss of 146 uma (M-146), producing a fragment ion at *m/z* 289.1005, due to the loss of catechin monomer unit, and they were identified as catechin deoxyhexose isomers, according to de Souza Mesquita et al. [38], who found the same compound in the mangrove tree. Peaks 19, 29 and 30, with molecular formula C_21_H_24_O_11_ and *m/z* 451.1262, showed a fragment ion at *m/z* 289.0896, and they were attributed to catechin glucoside isomers. A molecular ion at *m/z* 451 was also observed by García-Ruiz et al. [15] in freeze-dried and microencapsulated banana passion fruits; the major MS/MS fragment observed at *m/z* 289 indicated the loss of a hexose from the catechin moiety. Peaks 5, 11, 12, 13, 14 and 26 showed the same molecular formula, C_15_H_14_O_7_, with *m/z* 305.2595, and they were attributed to (epi)gallocatechin isomers. Peaks 42, 58 and 59, with molecular formula C_15_H_14_O_5_ and *m/z* 273, were attributed to (epi)fisetinidol isomers, according to Ballesteros-Vivas et al. [11]. Peaks 48, 52, 53, 64, 65 and 68 showed molecular formula C_21_H_24_O_10_ and *m/z* 435, with a characteristic fragment ion at *m/z* 273, corresponding to the glucosidic form of (epi)afzelechin, as also reported by García-Ruiz et al. [15]. Peaks 8, 25, 44 and 61, with molecular formula C_27_H_30_O_16_ and *m/z* 609, showed two fragment ions at *m/z* 435 and 273, and they were identified as (epi)azfelechin glucoside derivatives, as reported by García-Ruiz et al. [15]. Peak 18, at *m/z* 329.0892 and molecular formula C_17_H_14_O_7_, was tentatively identified as kaempferol-methoxy-methyl ether, as already described by Song et al. in another *Passiflora* fruit [39]. Proanthocyanidins consist of oligomeric forms sharing the flavan-3-ol monomers (epi)catechin, (epi)gallocatechin, (epi)azfelechin or (epi)fisetinidol. Peaks 15, 21, 32, 39, and 40, with molecular formula C_30_H_26_O_13_ and *m/z* 593, were identified as (epi)gallocatechin-(epi)catechin isomers; several isomers of these compounds have also been found by Ballesteros-Vivas et al. [11] in banana passion fruit seeds. As expected, all these peaks showed the presence of a fragment at *m/z* 289, corresponding to the catechin monomer. Peaks 20 and 75, with molecular formula C_30_H_24_O_12_ and *m/z* 575, were assigned to the A type procyanidin dimer (epi)catechin-(epi)catechin. Besides this, peaks 24, 33 and 60, with molecular formula C_30_H_26_O_12_ and *m/z* 577, were attributed to the B type procyanidin dimer (epi)catechin-(epi)catechin, as previously reported by Ballesteros-Vivas et al. [11]. The flavan-3-ol subunits of B type procyanidins are connected by a single bond, whereas in A type procyanidins, an additional ether linkage between adjacent monomers occurs. Peaks 34, 49, 55, 57, 66, 67 and 71, at *m/z* 561 and molecular formula C_30_H_26_O_11_, were tentatively identified as trihydroxy(iso)flavanol-(epi)catechin isomers (B type linkage). García-Ruiz et al. [15] and Verardo et al. [40] observed this compound in banana passion fruit and buckwheat, respectively; the authors assigned these peaks to a propelargonidin dimer composed of (epi)azfelechin-(epi)catechin. The same molecular ion at *m/z* 561 was identified as (epi)fisetinidol-(epi)catechin by Ballesteros-Vivas et al. [11]. This choice was explained by the presence of the fragment ion at *m/z* 409 (with a loss M-152), due to the typical retro-Diels–Alder (RDA) reaction of flavonoids [41]. Peak 72, with molecular formula C_45_H_38_O_16_ and *m/z* 833.2046, was assigned to the trimer (epi)azfelechin-(epi)azfelechin-(epi)catechin by Verardo et al. [40], whereas it was identified as (epi)fisetinidol-(epi)fisetinidol-(epi)catechin by Ballesteros-Vivas et al. [11]. Finally, another procyanidin trimer was attributed to peak 22, according to the findings of García-Ruiz et al. [15]. 

The peaks observed in the last part of the chromatogram (minutes 18–20) were tentatively identified as glucopyranoside, galactopyranoside and mannopyranoside derivatives; moreover, lactones and fatty acids were also observed. However, this was not the aim of the present study, so they will not be discussed in this paper. 

### 3.2. Free and Bound Phenolic Compounds Content and Their In Vitro Antioxidant and Hypoglycemic Activity

The present study quantified free (FPC) and bound (BPC) phenolic compounds in banana passion fruit, following the HPLC-ESI-TOF-MS analyses. In Table 3, the absolute and relative content levels of all free phenolic compounds identified are reported.

Banana passion fruit showed a total free phenolic compound content of 1468 mg 100 g^−1^ d.m. (dry matter).

Bound phenolic compound content is reported in Table 4. The total BPC amount was 878 mg 100 g^−1^ d.m. The sum of 3,4-dihydroxybenzoic acid and syringic acid hexoside represented 75.7% of the total bound fraction.

**Figure 1:**
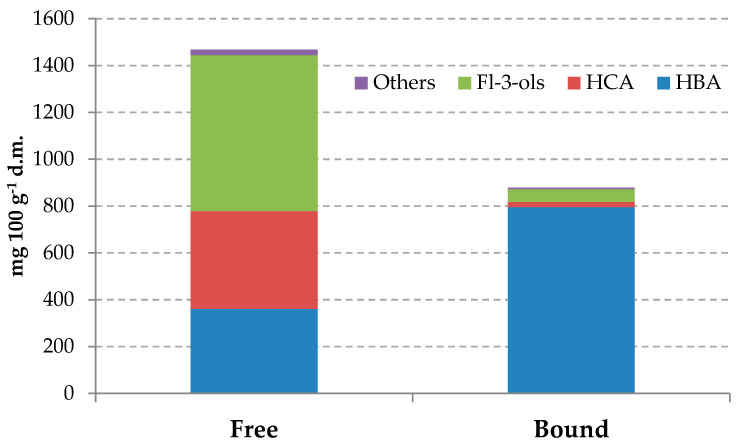
Distribution of phenolic compound classes in free and bound fraction. HBA, hydroxybenzoic acid derivatives; HCA, hydroxycinnamic acid derivatives; Fl-3-ols, flavan-3-ol derivatives.

Briefly, the total phenolic content (sum of free and bound phenolic fraction) was 2356 mg 100 g^−1^ d.m., as reported in Figure 1. FPC represented the most abundant fraction of compounds, amounting to 62.3% of the total content, whereas BPC constituted 33.7% of the total phenolic compounds. These results are in agreement with previous studies that found that phenolics in fruits were mainly present in soluble free form, at approximately 62%–96%, and only around 24% of total phenolics belonged to BPC [42]. Similar percentages were previously observed for banana and palm oil fruits, with 33.1% and 33.2% BPC in the total phenolic content, respectively [43].

**Figure 2:**
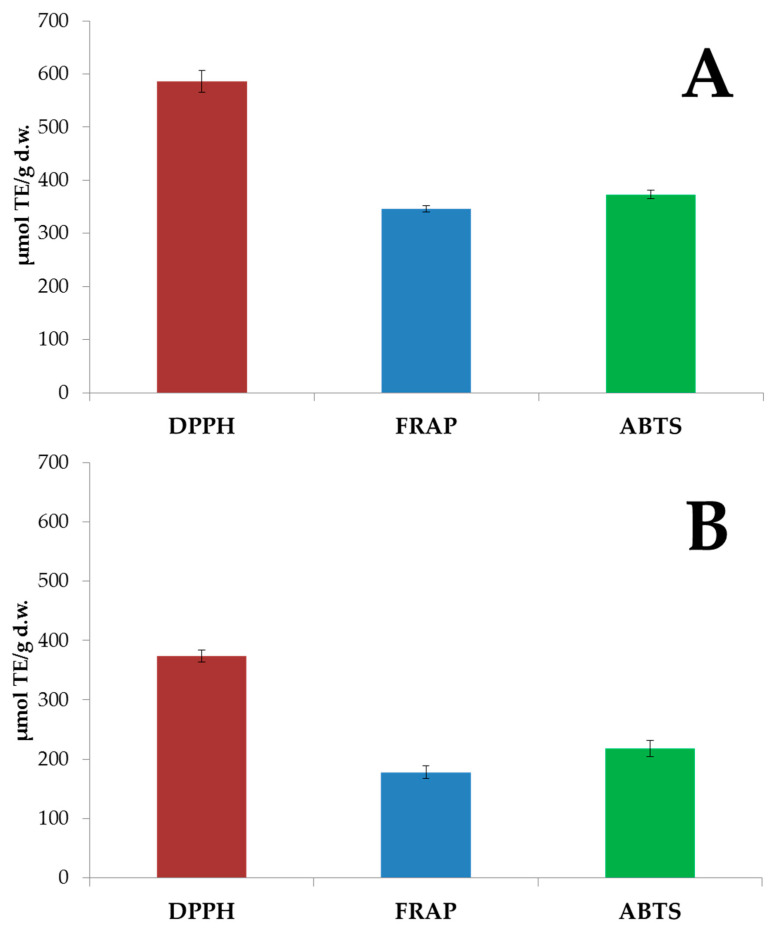
In vitro antioxidant capacities of free (**A**) and bound (**B**) phenolic compounds of banana passion fruit pulp.

The in vitro antioxidant capacities of free and bound phenolic extracts of banana passion fruit, evaluated by three different assays, are shown in Figure 2.

IC50 values of α-amylase inhibition were 20.8 ± 0.9 and 61.9 ± 1.2 µg/mL in FPC and BPC extracts, respectively.

## 4. Discussion

In the literature, several works have quantified the phenolic content of *Passiflora* species, with a particular focus on *Passiflora edulis* (passion fruit) and tropical fruits in general. Most of these works have determined the total phenolic, total flavonoid and total anthocyanin contents by spectrophotometric techniques. Septembre-Malaterre et al. [29] reported passion fruit as the richest source of phenolic compounds, compared with litchi, banana, pineapple, mango and papaya. Da Silva et al. [44] found 34.8 mg kg^−1^ d.m. of total anthocyanins and 603.7 mg kg^−1^ d.m. of total yellow flavonoids in passion fruit pulp, and similar values were also reported for its by-products; moreover, they determined a total phenolic content of 7651 mg gallic acid equivalent (GAE) kg^−1^ d.m., defining passion fruit as a good source of these compounds. A similar trend was reported by Ruiz-Torralba and co-workers [24], who noticed that *Passiflora edulis* f. flavicarpa fruit had the highest phenolic content of 52 fruits sold in Spain. Brat et al. [45], with the objective of creating a French database of the polyphenolic content of fruits and vegetables, reported 718 mg of GAE kg^−1^ fresh matter (f.m.) for passion fruit, and, considering a mean moisture value of 80%, this content could be expressed as 3590 mg of GAE kg^−1^ d.m. Similar values were also found by Vasco et al. [3] for passion fruit, whereas much higher contents were detected for banana passion fruit, which, with 10,100 mg of GAE kg^−1^ f.m., was included as a “high level phenolic compounds” fruit, together with Andean blackberry and Capulí cherry peel. Again, Contreras-Calderón et al. [16] found for banana passion fruit the highest phenolic content, with a total of 6350 mg of GAE kg^−1^ f.m. In the same work, another banana passion fruit of the genus *Passiflora tarminiana* was taken into account, which was found to be even richer in phenolics (10,180 mg of GAE kg^−1^ f.m.). However, they analyzed pulp and seeds together, and it is important to highlight that seeds contain higher amounts of phenolic compounds than pulp.

Hydroxycinnamic acids and their derivatives constituted 28.4% of the total amount of FPC; in particular, dicaffeoylquinic acid derivatives were the main compounds (275 mg 100 g^−1^ d.m.). Meinhart et al. [46] investigated the content of dicaffeoylquinic acids in 64 fruits consumed in Brazil, showing that passion fruit presented the highest content of 3,5-dicaffeoylquinic acid, reaching 312 mg kg^−1^ d.m. in one sample. Other authors [26] found 14 mg of 3,5-dicaffeoylquinic acid per 100 mL of passion fruit juice.

The total quantity of flavan-3-ols in the FPC extract was 667mg 100 g^−1^ d.m. The most abundant compound was an isomer of (epi)azfelechin glucoside, followed by the monomer unit (epi)fisetinidol. These findings agree with García-Ruiz et al. [15], who indicated flavan-3-ols as the major constituents of the phenolic fraction of banana passion fruit pulp, after freeze-drying; the authors indicated (epi)azfelechin and its glucoside derivatives as the most abundant compounds among this class of phenolics.

FPC and BPC were mainly composed by phenolic acids (53% and 93.1% of the total amount, respectively). Flavan-3-ols were mainly present in the FPC extract, representing 45.4% of total FPC; contrarily, they contributed only 6.3% of the bound phenolic fraction. Very low amounts were observed for flavonols and flavone derivatives, representing 1.3% and 1.1% of FPC and BPC, respectively.

The antioxidant capacity of free phenolic compounds (Figure 2A) measured by the DPPH assay was 587 µmol TE/g (d.w.) of the sample. This result strongly agrees with that reported by Garcia-Ruiz et al. [15]. A lower value was found by Ruiz-Torralba and co-workers [24]. However, as reported by Garcia-Ruiz et al. [15], variability from 0.9 to 608 µmol TE/g (d.w.) was previously obtained in banana passion fruit. The antioxidant capacity tested by the FRAP assay was 346.2 µmol TE/g (d.w.); this value is in the same order of magnitude as that obtained by Contreras-Calderón et al. [16] and higher than that obtained by Ruiz-Torralba et al. [24]. Finally, the ABTS assay reported an antioxidant capacity of 373.1 µmol TE/g (d.w.); this value agrees with that obtained by Contreras-Calderón et al. [16], while a higher value was found by Simirgiotis and co-workers [12]. Generally, the present data confirmed that the antioxidant capacity of banana passion fruit is higher than that obtained in other fruits [15,16,24,47]. In fact, according to Garcia-Ruiz et al. [15], banana passion fruit reported an antioxidant capacity, measured by DPPH, that was more than 10 times higher than that reported for *Physalis peruviana* and *Carica papaya*. Comparing our data with the results reported by Ruiz-Torralba et al. [24] for other tropical fruits, banana passion fruit showed DPPH values from 11 to 66 times higher than starfruit, cherimoya, mango, maracuya and pitaya; similar results were also found for the ABTS (2.5–17.6 times higher in banana passion fruit) and FRAP (4.7–39.4 times higher in banana passion fruit) assays. ABTS and DPPH values which were more than 300 times lower than banana passion fruit were described by Beserra-Almeida et al. [47] for Brazilian fruits such as jackfruit, mangaba, murici, papaya, pineapple, sapodilla, soursop, sweetsop, tamarind and umbu.

Figure 2B shows the antioxidant capacities of the bound phenolic extracts. As expected, the values obtained for bound phenolic extracts were lower than those measured for free phenolics. The DPPH and ABTS values obtained in this work were 6.7 and 2.2 times higher than those obtained in araticum fruit pulp [48]. Moreover, the DPPH and FRAP results were higher than those reported for papaya fruit [49].

Pearson’s correlations between phenolic content and antioxidant capacity assays are reported in Table 5.

As reported in Table 5, several positive correlations in the range of 0.9835–0.9987 were found. These data agreed with the results described by other authors [15,47]. Negative correlations were shown between hydroxybenzoic derivatives and antioxidant capacity assays. Positive correlations were also noticed between the different antioxidant capacity assays (*r* = 0.9903, *p* < 0.001 between DPPH and ABTS; *r* = 0.9847, *p* < 0.001 between DPPH and FRAP; *r* = 0.9914, *p* < 0.001 between ABTS and FRAP), confirming the suitability of the three assays to measure the antioxidant capacity in this fruit. Similar results were also noticed by other authors [16,47].

Inhibition analysis of α-amylase activity was conducted in order to corroborate the potential antihyperglycemic activity of phenolic compounds contained in *passiflora* passion fruit extracts. As reported by several authors [50,51], the inhibition of some key digestive enzymes, such as α-amylase, is a good strategy to treat or inhibit hyperglycemia. The highest α-amylase inhibition shown by the FPC extract could be justified by the higher content of phenolic compounds compared to the BPC extract. Moreover, the FPC extract contains high amounts of flavonoids that seem to be particularly involved in the hypoglycemic process [18]. Besides this, high correlations were found between α-amylase inhibition and total phenolic content (*r* = 0.9948, *p* < 0.001) and between α-amylase inhibition and total flavonoid content (*r* = 0.9999, *p* < 0.001). Data obtained for the FPC extract was very close to that obtained by Shanmugam et al. [52,53] in *Passiflora leschenaultia* and *Passiflora subpeltata* fruits. Higher values were found by Loizzo et al. [18] for free phenolic extracts of banana passion fruit and other *Passiflora* fruits; however, this discrepancy could be due to botanical or agronomical factors, and the different extraction methods for phenolic compounds.

## 5. Conclusions

This paper represents a further contribution to the investigation of the phytochemical composition of banana passion fruit, a lesser explored species found in Ecuador and other Latin American countries.

HPLC-ESI-TOF-MS was successfully applied in order to identify and quantify free and bound phenolic compounds extracted from banana passion fruit (*Passiflora mollissima*) pulp. By means of this analytical technique, 82 compounds were tentatively identified, and 80 of them were quantified. Free phenolic compounds represented the most abundant fraction of phenolics. The total phenolic acids (hydroxybenzoic plus hydroxycinnamic acid derivatives) were the highest concentrated phenolic group in both free and bound phenolic fractions. However, flavan-3-ols were the most concentrated compounds of the free phenolic fraction, considering the single classes. Moreover, the antioxidant capacities measured with three different assays showed that banana passion fruit presented a high antioxidant activity, significantly higher than in other fruits. These new data regarding free and bound phenolic identification and quantification and the evaluation of the antioxidant capacity of banana passion fruit may represent an incentive to promote the use of this fruit in food preparation. Indeed, this new information about banana passion fruit’s phenolic composition could also be used to characterize banana passion fruits from different countries and/or different food processes. Briefly, the high phenolic content and the antioxidant and antihyperglycemic capacity found in this fruit confirmed its potential as a functional fruit. However, in vitro assays are only explorative determinations; because of this, further in vivo analyses are necessary in order to confirm these data and to corroborate the bioaccessibility and bioavailability of the phenolic compounds present in banana passion fruit.

## Figures and Tables

**Table 1:** Soluble solids (° Brix), pH and acidity values.

Parameter	Result	Reference Method [22]
Soluble solids (° Brix)	11.42 ± 0.03	AOAC 932.12
pH	3.53 ± 0.02	AOAC 981.12
Acidity (g/100 g of citric acid equiv.)	1.08 ± 0.02	AOAC 942.15

**Table 2:** Phenolic compounds identified as free and bound forms in banana passion fruit pulp.

Peak N	Compound	RT (min)	Molecular Formula	Calculated Mass [M−H]^−^	Experimental Mass [M−H]^−^	Error (ppm)	mSigma	In Source Fragments
1	quinic acid	0.79	C_7_H_12_O_6_	191.0197	191.0268	8.8	14.2	
2	citric acid	0.94	C_6_H_8_O_7_	191.0197	191.027	8.2	12.4	
3	dicaffeoylquinic acid derivative1	1.48	C_22_H_28_O_14_	515.1406	515.1413	8.1	18.6	353.0976, 191.0267, 179.0674
4	dicaffeoylquinic acid derivative 2	1.86	C_22_H_28_O_14_	515.1406	515.1413	−1.2	19.4	353.0976, 191.0267, 179.0674
5	(epi)gallocatechin	2.01	C_15_H_14_O_7_	305.0667	305.0671	−4.1	24.1	191.0294
6	dicaffeoylquinic acid derivative 3	2.15	C_22_H_28_O_14_	515.1406	515.141	−0.3	24.5	353.0976, 191.0271, 179.0503
7	caffeoyltartaric acid derivative	2.38	C_22_H_24_O_15_	527.1042	527.105	−1.5	15.2	469.0998, 349.0535, 191.0285
8	(epi)-azfelechin glucoside derivative	2.95	C_27_H_30_O_16_	609.1402	609.1442	2.6	9.1	527.1062, 353.0854, 191.0272
9	3,4-dihydroxybenzoic acid (protocatechuic)	2.98	C_7_H_6_O_4_	153.0193	153.0152	−3.6	13	-
10	syringic acid	3.55	C_9_H_10_O_5_	197.0455	197.0415	−5.1	40.2	179.0342, 167.0405, 123.0528
11	(epi)gallocatechin	4.14	C_15_H_14_O_7_	305.0667	305.0669	−1.5	13.9	-
12	(epi)gallocatechin	4.32	C_15_H_14_O_7_	305.0667	305.0777	−3.0	16.4	219.1106, 191.0266
13	(epi)gallocatechin	4.37	C_15_H_14_O_7_	305.0667	305.0773	−3.0	16.9	219.0607, 191.0271
14	(epi)gallocatechin	4.48	C_15_H_14_O_7_	305.0667	305.0773	−2.9	15.8	219.0607, 191.0271
15	(epi)gallocatechin-(epi)catechin	4.72	C_30_H_26_O_13_	593.1301	593.1377	−7.2	13.1	527.1595, 353.0831, 191.0279
16	myricetin hexoside isomer	5.21	C_21_H_20_O_13_	479.0831	479.0881	−6.0	7.1	329.0988, 219.0590, 173.015
17	4-hydroxybenzoic acid	5.34	C_7_H_6_O_3_	137.0244	137.0297	−4.7	8.9	155.0405
18	Kaempferol-methoxy-methyl ether	5.68	C_17_H_14_O_7_	329.0878	329.0892	−8.8	16.8	-
19	catechin hexoside	6.10	C_21_H_24_O_11_	451.1246	451.1262	−5.8	13	365.1499, 289.0896
20	(epi)catechin-(epi)catechin	6.16	C_30_H_24_O_12_	575.1195	575.1176	−4.5	32.2	375.0857, 287.0674
21	(epi)gallocatechin-(epi)catechin	6.45	C_30_H_26_O_13_	593.1301	593.1386	−6.2	8.5	289.0786
22	procyanidin trimer	6.53	C_45_H_38_O_20_	897.1884	897.1867	−3.6	40.5	479.1002, 425.0999, 289.0788
23	syringic acid isomer	6.58	C_9_H_10_O_5_	197.0455	197.0429	−3.5	8.5	179.0428, 165.0259
24	(epi)catechin-(epi)catechin	6.66	C_30_H_26_O_12_	577.1351	577.1327	−5.4	8.7	425.1043, 289.0572
25	(epi)azfelechin glucoside derivative	6.77	C_27_H_30_O_16_	609.1402	609.1441	3.4	18.5	289.0815
26	(epi)gallocatechin	6.90	C_15_H_14_O_7_	305.0667	305.0666	−2.4	3.8	
27	catechin	7.15	C_15_H_14_O_6_	289.0718	289.0721	−3.6	2.2	
28	isoquercitrin	7.27	C_21_H_20_O_12_	463.0882	463.0841	−3.3	15.2	329.0981, 289.0823, 175.0313
29	(epi)catechin-glucoside	7.54	C_21_H_24_O_11_	451.1246	451.1293	−3.6	5.7	289.0811, 229.1064
30	(epi)catechin-glucoside	7.60	C_21_H_24_O_11_	451.1246	451.1284	−3.5	9	289.0789, 229.106
31	caffeic acid	7.74	C_9_H_8_O_4_	179.035	179.0311	−4.4	19.4	135.0506
32	(epi)gallocatechin-(epi)catechin	7.94	C_30_H_26_O_13_	593.1301	593.1393	−3.5	17.6	407.1109, 289.0777
33	(epi)catechin-(epi)catechin	8.04	C_30_H_26_O_12_	577.1351	577.1353	−2.9	10.8	451.1465, 327.1182, 289.1005
34	trihydroxy(iso)flavanol-(epi)catechin	8.76	C_30_H_26_O_11_	561.1402	561.1471	−3.1	8	409.1127, 289.1006
35	eriodictyol-*O*-hexoside	8.97	C_21_H_22_O_11_	449.1242	449.1234	−32.1	5.8	317.1382, 191.0213, 179.059
36	luteolin-6-C-glucoside (orientin)	8.99	C_21_H_20_O_11_	447.0933	447.0978	−3.4	9.9	431.1019, 377.1054
37	catechin deoxyhexose	9.08	C_21_H_24_O_10_	435.1297	435.123	−3.6	3.3	317.1349, 289.1005, 173.0148
38	catechin deoxyhexose	9.23	C_21_H_24_O_10_	435.1297	435.1228	−3.1	6.2	317.1156, 289.0993
39	(epi)gallocatechin-(epi)catechin	9.26	C_30_H_26_O_13_	593.1301	593.1318	−3.6	29.2	289.0815, 179.0423
40	(epi)gallocatechin-(epi)catechin	9.39	C_30_H_26_O_13_	593.1301	593.1398	−3.3	12.9	289.0843, 179.0432
41	parasorboside	9.41	C_12_H_20_O_8_	291.1085	291.1094	−3.2	4.8	173.0161
42	(epi)fisetinidol	9.51	C_15_H_14_O_5_	273.0768	273.0766	2.1	10.3	149.0343
43	lucenin-2,4-methyl ether (diosmetin 6,8-di-C-hexoside)	9.91	C_28_H_32_O_16_	623.1618	623.1646	3.5	2.1	567.1891, 381.1902
44	(epi)azfelechin glucoside derivative	10.03	C_27_H_30_O_16_	609.1402	609.1457	−3.2	12.7	435.1368, 273.0773
45	digalloylglucose isomer	10.03	C_20_H_20_O_14_	483.078	483.0931	−6.6	19.6	377.1008, 321.0748
46	digalloylglucose isomer	10.22	C_20_H_20_O_14_	483.078	483.0934	−6.7	15.7	377.1008, 321.0748
47	eriodictyol-*O*-hexoside	10.26	C_21_H_22_O_11_	449.1289	449.1235	−3.5	10	173.0162
48	(epi)azfelechin glucoside	10.38	C_21_H_24_O_10_	435.1297	435.1237	−3.2	8.7	273.0888
49	trihydroxy(iso)flavanol-(epi)catechin	10.78	C_30_H_26_O_11_	561.1402	561.1479	−3.6	6.5	449.1333, 289.1656
50	eriodictyol	11.08	C_15_H_12_O_6_	287.0561	287.0566	−3.4	9.3	-
51	daidzin	11.18	C_21_H_20_O_9_	415.1035	415.1068	−2.2	15.8	359.0897, 281.077
52	(epi)azfelechin glucoside	11.23	C_21_H_24_O_10_	435.1297	435.1236	−3.9	6.9	273.0867
53	(epi)azfelechin glucoside	11.32	C_21_H_24_O_10_	435.1297	435.1233	−3.3	5.6	273.1089
54	lucenin-2,4-methyl ether (diosmetin 6,8-di-C-hexoside)	11.40	C_28_H_32_O_16_	623.1618	623.1641	2.3	17.5	463.1415, 435.1502
55	trihydroxy(iso)flavanol-(epi)catechin	11.59	C_30_H_26_O_11_	561.1402	561.1467	−2.4	10.1	479.0994, 439.1923, 409.1001
56	baicalin	11.69	C_21_H_18_O_11_	445.0776	445.0716	−3.3	7.2	891.1876, 291.1229, 173.0167
57	trihydroxy(iso)flavanol-(epi)catechin	11.72	C_30_H_26_O_11_	561.1402	561.1476	−3.3	27.7	483.1149, 435.144, 313.0921
58	(epi)fisetinidol	11.85	C_15_H_14_O_5_	273.0768	273.0768	−3.3	7.8	149.0291, 123.0508
59	(epi)fisetinidol	12.00	C_15_H_14_O_5_	273.0768	273.0763	−3.6	13.4	173.0142, 149.0276, 123.0383
60	(epi)catechin-(epi)catechin	12.00	C_30_H_26_O_12_	577.1351	577.1335	−3.4	35.6	447.1115, 273.0863
61	(epi)azfelechin glucoside derivative	12.21	C_27_H_30_O_16_	609.1402	609.1444	−3.1	9.3	561.1585, 435.137, 273.0849
62	isorhamnetin-*O*-dihexoside	12.41	C_28_H_32_O_17_	639.1203	639.1273	−4.9	11.5	561.1618, 477.1199
63	rosmarinic acid	12.47	C_18_H_16_O_8_	359.0772	359.0795	−4.3	18.3	289.0587, 217.1178
64	(epi)azfelechin glucoside	12.74	C_21_H_24_O_10_	435.1297	435.1231	−3.9	7.2	273.0879
65	(epi)azfelechin glucoside	12.87	C_21_H_24_O_10_	435.1297	435.1235	−3.8	5.1	273.0877
66	trihydroxy(iso)flavanol-(epi)catechin	12.99	C_30_H_26_O_11_	561.1402	561.1483	−3.2	18.1	345.0787, 251.0658
67	trihydroxy(iso)flavanol-(epi)catechin	13.16	C_30_H_26_O_11_	561.1402	561.1478	−3.3	16.4	449.164, 435.1421
68	(epi)azfelechin glucoside	13.21	C_21_H_24_O_10_	435.1297	435.1234	−3.5	12.1	273.0846
69	syringic acid hexoside	13.24	C_15_H_20_O_10_	359.0984	359.0992	2.6	11.3	251.0642
70	phloretin dihexoside	13.60	C_27_H_33_O_15_	597.1825	597.1818	−3.3	6.3	477.1434, 387.2049
71	trihydroxy(iso)flavanol-(epi)catechin	14.03	C_30_H_26_O_11_	561.1402	561.1481	−3.8	19.6	447.1132, 413.1034
72	(epi)azfelechin-(epi)azfelechin -(epi)catechin	14.47	C_45_H_38_O_16_	833.2087	833.2046	−3.2	14.5	561.1625, 429.2246, 331.1875, 179.0626
73	diosmetin--rutinoside	14.65	C_28_H_32_O_15_	607.1668	607.1625	7.1	13.5	411.1149, 343.0861
74	trihydroxyflavanone dimer	14.67	C_30_H_26_O_10_	545.1453	545.1426	−3.7	16.1	447.1091, 431.2326, 173.0151
75	(epi)catechin-(epi)catechin	15.47	C_30_H_24_O_12_	575.1195	575.1188	−3.5	24.9	378.1656, 285.0525
76	durantin A	15.54	C_22_H_20_O_9_	427.1035	427.1048	−2.6	12.6	341.0792, 249.0385
77	diosmetin-rutinoside	16.12	C_28_H_32_O_15_	607.1668	607.1647	3.6	17.3	467.1121, 341.0817
78	2,6-dihydroxy-3-methyl-4-*O*-(6’’-*O*-galloyl-β-d-glucopyranosyl)-benzophenone	16.64	C_27_H_26_O_13_	557.1301	557.1256	7.9	16.4	521.276, 431.2423, 269.1844
79	caffeoylglucaric acid derivative	17.56	C_24_H_52_O_14_	563.3284	563.3261	4.2	12.9	547.2124, 471.2775
80	myricetin derivative	18.35	C_30_H_60_O_20_	739.3605	739.363	−3.4	19.8	565.3407, 489.2849
81	methylsyringin	18.47	C_18_H_26_O_9_	385.1504	385.1522	2.3	11.8	295.1442, 265.1188
82	(epi)azfelechin glucoside derivative	19.00	C_27_H_52_O_12_	567.3386	567.3376	−3.4	2.2	475.3059

**Table 3:** Quantification of free phenolic compounds (FPC) of banana passion fruit pulp (mg 100 g^−1^ d.m.).

Peak N	Compound	FPC	% of the total FPC
		(mg 100 g^−1^ d.m.)	
	**Hydroxybenzoic acid derivatives**		
81	methylsyringin	361.51 ± 0.89	24.6
	**Hydroxycinnamic acid derivatives**		
3	dicaffeoylquinic acid	144.95 ± 0.63	9.9
4	dicaffeoylquinic acid	86.63 ± 0.27	5.9
6	dicaffeoylquinic acid	43.64 ± 0.51	3.0
7	caffeoyltartaric acid derivative	93.08 ± 0.67	6.3
79	caffeoylglucaric acid derivative	48.39 ± 0.29	3.3
	**Flavonols and flavone derivatives**		
16	myricetin hexoside isomer	2.08 ± 0.21	0.1
18	kaempferol-methoxy-methyl ether	0.67 ± 0.09	0.05
28	isoquercitrin	0.98 ± 0.01	0.1
36	eriodictyol-*O*-hexoside	3.67 ± 0.10	0.2
43	lucenin-2,4-methyl ether (diosmetin 6,8-di-C-hexoside)	1.09 ± 0.05	0.1
47	eriodictyol-*O*-hexoside	1.46 ± 0.02	0.1
50	eriodictyol	1.72 ± 0.02	0.1
54	lucenin-2,4-methyl ether (diosmetin 6,8-di-C-hexoside)	0.56 ± 0.01	0.04
56	baicalin	1.60 ± 0.03	0.1
62	isorhamnetin-*O*-dihexoside	1.43 ± 0.12	0.1
70	naringenin hexose derivate	2.03 ± 0.23	0.1
74	trihydroxyflavanone dimer	0.90 ± 0.01	0.1
80	myricetin derivative	5.09 ± 0.16	0.3
	**Flavan-3-ol derivatives**		
5	(epi)gallocatechin	5.66 ± 0.11	0.4
8	(epi)azfelechin glucoside derivative	105.20 ± 1.38	7.2
11	(epi)gallocatechin	3.46 ± 0.64	0.2
12	(epi)gallocatechin	24.81 ± 0.60	1.7
13	(epi)gallocatechin	26.34 ± 0.41	1.8
14	(epi)gallocatechin	1.42 ± 0.09	0.1
19	catechin hexoside	2.44 ± 0.19	0.2
27	catechin	9.61 ± 0.53	0.7
29	(epi)catechin-glucoside	2.74 ± 0.11	0.2
30	(epi)catechin-glucoside	5.66 ± 0.08	0.4
37	catechin deoxyhexose	7.26 ± 0.37	0.5
38	catechin deoxyhexose	33.42 ± 0.70	2.3
44	(epi)azfelechin glucoside derivative	3.36 ± 0.05	0.2
48	(epi)azfelechin glucoside	52.28 ± 0.99	3.6
52	(epi)azfelechin glucoside	8.08 ± 0.30	0.6
53	(epi)azfelechin glucoside	2.90 ± 0.06	0.2
58	(epi)fisetinidol	76.74 ± 1.03	5.2
59	(epi)fisetinidol	2.96 ± 0.44	0.2
61	(epi)azfelechin glucoside derivative	1.33 ± 0.01	0.1
64	(epi)azfelechin glucoside	6.02 ± 0.20	0.4
65	(epi)azfelechin glucoside	38.56 ± 1.06	2.6
68	(epi)azfelechin glucoside	9.36 ± 0.14	0.6
82	(epi)azfelechin glucoside derivative	91.18 ± 1.51	6.2
15	(epi)gallocatechin-(epi)catechin	64.45 ± 2.02	4.4
21	(epi)gallocatechin-(epi)catechin	3.58 ± 0.07	0.2
22	procyanidin trimer	1.29 ± 0.03	0.1
24	(epi)-catechin-(epi)catechin	10.20 ± 0.80	0.7
33	(epi)-catechin-(epi)catechin	2.53 ± 0.05	0.2
34	trihydroxy(iso)flavanol-(epi)catechin	5.95 ± 0.27	0.4
49	trihydroxy(iso)flavanol-(epi)catechin	45.48 ± 0.39	3.1
55	trihydroxy(iso)flavanol-(epi)catechin	4.92 ± 0.08	0.3
67	trihydroxy(iso)flavanol-(epi)catechin	3.94 ± 0.10	0.3
72	(epi)azfelechin-(epi)azfelechin-(epi)catechin	3.57 ± 0.04	0.2

**Table 4:** Quantification of bound phenolic compounds (BPC) of banana passion fruit pulp (mg 100 g^−1^ d.m.).

Peak N	Compound	BPC	% of the total BPC
		(mg 100 g^−1^ d.m.)	
	**Hydroxybenzoic acid derivatives**		
9	3,4-dihydroxybenzoic acid	327.62 ± 2.29	37.3
10	syringic acid	60.65 ± 1.13	6.9
17	4-hydroxybenzoic acid	7.63 ± 1.07	0.9
23	syringic acid	61.68 ± 1.39	7.0
69	syringic acid hexoside	337.57 ± 2.98	38.4
	**Hydroxicinnamic acid derivatives**		
31	caffeic acid	1.38 ± 0.06	0.2
45	digalloylglucose isomer	6.16 ± 0.14	0.7
46	digalloylglucose isomer	10.25 ± 0.02	1.2
63	rosmarinic acid	4.84 ± 0.08	0.6
	**Flavonol and flavone derivatives**		
36	luteolin-6-C-glucoside (orientin)	3.09 ± 0.07	0.4
51	daidzin	0.54 ± 0.02	0.1
73	diosmetin-7-*O*-rutinoside	0.57 ± 0.02	0.1
76	durantin A	0.44 ± 0.06	0.1
77	diosmetin-7-*O*-rutinoside	1.09 ± 0.03	0.1
	**Flavan-3-ol derivatives**		
12	(epi)gallocatechin	7.21 ± 0.18	0.8
13	(epi)gallocatechin	3.30 ± 0.10	0.4
25	(epi)azfelechin glucoside derivative	1.32 ± 0.05	0.2
26	(epi)gallocatechin	1.07 ± 0.01	0.1
27	catechin	9.88 ± 0.40	1.1
42	(epi)fisetinidol	7.17 ± 0.27	0.8
48	(epi)azfelechin glucoside	2.63 ± 0.19	0.3
59	(epi)fisetinidol	1.97 ± 0.11	0.2
64	(epi)azfelechin glucoside	1.35 ± 0.07	0.2
65	(epi)azfelechin glucoside	2.50 ± 0.01	0.3
68	(epi)azfelechin glucoside	3.35 ± 0.13	0.4
20	(epi)catechin-(epi)catechin	1.85 ± 0.02	0.2
32	(epi)gallocatechin-(epi)catechin	0.29 ± 0.01	0.03
39	(epi)gallocatechin-(epi)catechin	2.05 ± 0.08	0.2
40	(epi)gallocatechin-(epi)catechin	3.03 ± 0.04	0.3
57	trihydroxy(iso)flavanol-(epi)catechin	0.85 ± 0.09	0.1
60	(epi)-catechin-(epi)catechin	1.89 ± 0.18	0.2
66	trihydroxy(iso)flavanol-(epi)catechin	1.34 ± 0.06	0.2
71	trihydroxy(iso)flavanol-(epi)catechin	1.19 ± 0.12	0.1
75	(epi)catechin-(epi)catechin	0.73 ± 0.05	0.1

**Table 5:** Correlation coefficients (*p* < 0.001) obtained between phenolic content and antioxidant assays in banana passion fruit.

Compounds	DPPH	ABTS	FRAP
Total phenolic compounds	0.9987	0.9902	0.9835
HBA	−0.9913	−0.9961	−0.9925
HCA	0.9930	0.9965	0.9938
Flavan-3-ols	0.9931	0.9966	0.9936
Others	0.9969	0.9963	0.9919

## Supplementary Materials

The following are available online at https://www.mdpi.com/2076-3921/9/7/628/s1, Figure S1: Base peak chromatogram of free (FPC) and bound (BPC) phenolic compounds in banana passion fruit obtained by HPLC-ESI-TOF-MS.
