# Supplementary material for: New Advances in the Determination of Free and Bound Phenolic Compounds of Banana Passion Fruit Pulp (Passiflora tripartita, var. Mollissima (Kunth) L.H. Bailey) and Their In Vitro Antioxidant and Hypoglycemic Capacities
Source: Antioxidants (Basel). 2020 Jul 17;9(7):628. doi: 10.3390/antiox9070628 (PMC7402170; doi:10.3390/antiox9070628)

Supplementary Figure. Base Peak Chromatogram of free (FPC) and bound (BPC) phenolic compounds in banana passion fruit obtained by HPLC-ESI-TOF-MS

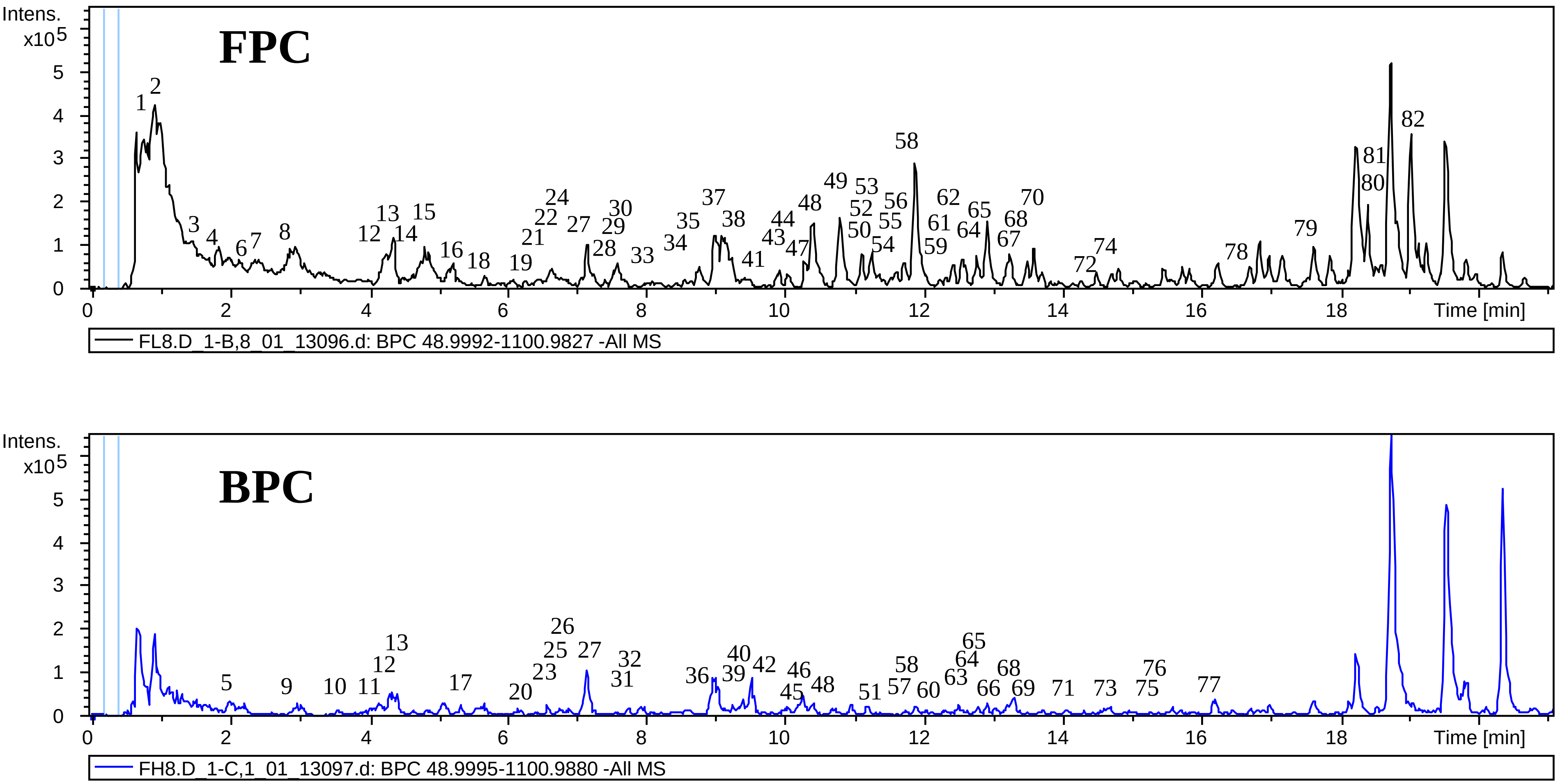

Supplement: Supplementary file 1 [file antioxidants-09-00628-s001.pdf]
